# Augmenting phyto-chemical and phyto-mineral profiling of moringa leaf extract: A contrastive study of solid-liquid extraction methodologies

**DOI:** 10.1016/j.heliyon.2024.e40909

**Published:** 2024-12-04

**Authors:** Arumugam Thangaiah, Sandeep Gunalan, Premalakshmi Velu, Dheebisha Chandirasekaran, Aruliah Rajasekar, Mohamad S. AlSalhi, Sandhanasamy Devanesan, Tabarak Malik

**Affiliations:** a Department of Biotechnology, Thiruvalluvar University, Vellore District, Tamil Nadu, India; b Department of Horticulture, Palar Agricultural College, Melpatti, Vellore District, Tamil Nadu, India; c Department of Horticulture, V.O.Chidambaranar Agricultural College and Research Institute, TNAU, Killikulam, Tamil Nadu, India; d Department of Horticulture, Adhiparasakthi Agricultural College, Kalavai, Ranipet District, Tamil Nadu, India; e Adjunct Faculty, Department of Prosthodontics, Saveetha Dental College& Hospital, Chennai, 600 077, Tamil Nadu, India; f Environmental Molecular Microbiology Research Laboratory, Department of Biotechnology, Thiruvalluvar University, Vellore, 632115, India; g Department of Physics and Astronomy, College of Science, King Saud University, P. O. Box 2455, Riyadh, 11451, Saudi Arabia; h Department of Biomedical Sciences, Institute of Health, Jimma University, Ethiopia; i Adjunct Faculty, Division of Research and Development, Lovely Professional University, Phagwara 144411 India

**Keywords:** Malnutrition, *Moringa oleifera*, Novel methodologies, Phyto-minerals, Solid-Liquid extraction, Ultrasound-assisted extraction

## Abstract

The global food system is plagued by legitimacy and stability issues due to climate and ecosystem disruptions, contributing to widespread malnutrition. A significant portion of the global population experiences undernourishment, overweight, and micronutrient deficiencies from unhealthy diets. Addressing these challenges necessitates regular consumption of essential nutrients from plant sources. Among various crops, *Moringa oleifera* leaves are highly nutritious, offering essential vitamins, minerals, and medicinal properties. Whence research was conducted to inquire the proximate phytochemical composition and extraction efficiency of moringa leaf extracts across four extraction methods: maceration (E_1_), Soxhlet (E_2_), ultrasound-assisted extraction (UAE) (E_3_), and microwave-assisted extraction (MAE) (E_4_) using 70 % hydro-ethanolic solvent. The study detected the presence of phytocompounds and phytonutrients at higher levels in UAE and MAE extracts. Recovery yield was highest for UAE (21.81 ± 0.32 %) than conventional extraction methods (6.24 ± 0.08 %). Both advanced extraction methods resulted in higher TPC (148.86 ± 1.92 mg GAE/g and 137.65 ± 4.49 mg GAE/g, respectively) and TFC (23.18 ± 0.44 mgQE/g and 22.12 ± 0.61 mg QE/g, respectively). Protein and ascorbic acid contents followed a similar trend, with UAE achieving 148.66 ± 3.74 mg/ml and 620.25 ± 1.42 mg/100g, respectively. Antioxidant activity measured by DPPH assay was highest in UAE (86.25 ± 1.58 %) along with the lowest IC_50_ values (11.62 ± 1.58 μg/ml) in UAE. Furthermore, ICP-OES analysis revealed higher concentrations of essential phyto-minerals in moringa leaf extracts. Moreover, SEM analysis revealed significant morphological disruptions in the leaf samples, correlating with higher phytomolecules recovery. The outcome of the research is that novel extraction methods significantly enhanced the extraction efficiency and quality of bioactive compounds from moringa leaves, suggesting their potential in the development of nutraceutical and biofortified food products to expunge malnutrition.

## Highlights

• Moringa leaves are rich source of phyto-nutrients & phyto-minerals.
• Ultrasonication extracted qualitative biomolecules from moringa leaves.
• ICP-OES analysis revealed the presence of phytominerals.
• Moringa leaf extracts are potential source of nutraceutical supplements.
• Moringa leaf extracts can be utilized to biofortify dietary supplements.

## Graphical abstract

**Figure:**
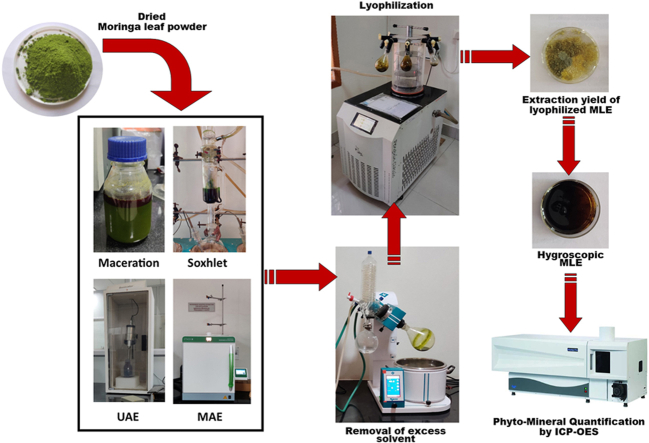

## 1 Introduction

The current global food system is not legitimate and it gets affected with its stability due to resilience in climate and ecosystem. A large portion of the world's population is found to be suffering from malnutrition which includes undernourishment, overweight and micronutrient deficiencies due to unhealthy diets [1]. Nutrient deficiency is the red flagged concern in various developing countries, which leads to serious health ailments. As humans are reported to have various malnutrition problems during their growth, those problems can be resolved by regular intake of essential nutrients, vitamins and minerals from plant sources [2].

Sustainable agriculture and improved agricultural technologies increased the production of fruits and vegetables in the world. It is reported that Plant-based diets are reducing the mortality rate and other contagious diseases [3]. Plant-based diets include various forms such as vegan, flexitarian, lacto-ovo-vegetarian and semi-vegetarian diets. Production of plants through intensive cultivation and acquiring them for consumption paves the way for human beings to intake recommended nutrients on a daily basis. For centuries, phytonutrients and phytochemicals, played crucial role in maintaining homeostasis across the world. These naturally occurring compounds are extracted through various extraction methods by highlighting their medicinal, nutraceutical and food fortification values [4].

Among various horticultural crops that are preferred for cooking and salads, moringa leaves were found to have extremely high quantity of all phytonutrients and other biochemical. Moringa leaves are highly nutritious and also found to have enormous medicinal properties along with the benefits of supplying plant-based vital nutrients for human beings. Moringa leaves are considered to contain more protein and Sulfur-containing amino acids, more vitamin C than citrus, more vitamin A and calcium than carrots and milk, respectively. Moreover, moringa leaves are also found to contain 9 folds of iron than in spinach, more potassium than bananas and 4 folds of fiber than oats [2]. Moringa could be an alternative source of antioxidants, antibiotics, vitamins and minerals. The diverse nutrient profile of *Moringa oleifera* leaves makes them an excellent addition to the regular diet for combating malnutrition.

*Moringa oleifera Lam.,* commonly referred to as the “Drumstick tree,” is believed to have originated in India, particularly in the sub-Himalayan regions. It belongs to the monogenous family known as Moringaceae and around 13 species have been reported in this family under the genus Moringa [5]. Moringa leaves not only found with various nutrients, phytocompounds and also it is reported with various medicinal properties such as antitumor, antipyretic, antiepileptic, anti-inflammatory, antiulcer, anti-diabetic, antioxidant, antifungal, and antibacterial [6]. To utilize the nutrients available in the moringa leaves effectively against malnutrition through biofortification, a research trial was conducted by extracting the phytocompounds from the leaf matrix through conventional and non-conventional extraction methods. Extraction is a fundamental process followed to extract natural compounds from the plant source, to develop and enhance cost effective bio-compounds which might be a better source for pharmaceutical, nutraceutical, cosmetics and food-based industries. Different extraction methods are show significance in terms of efficiency, selectivity and environmental impact. A comparative analysis was performed on extraction techniques such as maceration, Soxhlet extraction, ultrasound-assisted extraction, and microwave-assisted extraction to assess the qualitative and quantitative characteristics of *Moringa oleifera* leaf extracts.

## 2 Materials and methods

### 2.1 Plant material

The moringa leaves of variety PKM 1, utilized in this research study was sourced from the organic fields of the Horticultural College and Research Institute in Periyakulam, Theni District, Tamil Nadu, and India. Moringa leaves were harvested at 45-day intervals and dried using a cabinet solar dryer at 40 ± 2 °C for 4 h. Following drying, the dried moringa leaves were pulverized using a commercial-grade pulverizer and sieved through a 200 μm mesh sieve. The pulverized leaves were then stored under refrigerated and dark conditions at 4 °C for subsequent analysis.

### 2.2 Methods

#### 2.2.1 Extraction procedure

All the extraction methods were conducted with sample: solvent ratio of 1:10 for better recovery of moringa leaf extract (MLE). The powdered moringa leaves were used for extraction under a 70 % hydroethanolic green solvent phase system based on the standardized extraction methodologies proposed by Refs. [7,8]. Consistency in the sample-to-solvent mixture ratio was upheld across all extraction methods, and each corresponding mixture underwent various extraction techniques.

##### 2.2.1.1 Maceration extraction method

Tradition soaking method is the oldest Ayurvedic method by soaking plant material in the solvent system for a prolonged period. Dried powdered moringa leaves and 70 % ethanol were mixed in a conical flask at a ratio 1:10 [7]. The samples were placed in a dark environment for 24 h with sporadic shaking and filtered to collect the supernatant.

##### 2.2.1.2 Soxhlet extraction method

Soxhlet extraction was performed by placing the powdered moringa leaf samples in the extraction chamber contained in a Soxhlet glass thimble employing 70 % hydro-ethanol in 1:10 ratio at 40 ± 5 °C for 8 cycles or 16 h of siphoning. Extracted MLE were concentrated by vacuum rotovap at 40 ± 2 °C using IKA RV 3 Pro V Complete rotary evaporator, frozen at −80 °C and lyophilized using Innova INOFD 12S, Innova Bio-Meditech, China. Freeze-dried MLE was stored at −4 °C, under refrigerated conditions for further analysis.

##### 2.2.1.3 Ultrasound-assisted extraction method

Powdered moringa leaf samples were mixed with ethanol/water 70 % v/v solvent system at 1:10 sample: solvent ratio using full wave solid probe sonicator accustomed with 254 mm long Ti-Al alloy (Sonics VCX 1500 Series, Mfd. by Sonics & Materials Inc., USA). The sonicator and temperature probe were immersed in the moringa leaf-solvent mixture, which was placed in an ice bath throughout the procedure to ensure the extraction temperature did not exceed 30 °C, preventing degradation of biomolecules. The extraction system was maintained with 70 % amplitude energy and a pulsing cycle on-off for 5 s according to the optimized protocol of [9]. After sonication, the samples underwent centrifugation at 10000 rpm and 15 ± 2 °C for 10 min using a CR22N refrigerated centrifuge (Eppendorf Himac Technologies Co., Ltd., Japan). The moringa supernatant was collected, concentrated using vacuum rotovap (RV 3 Pro V, IKA India, Pvt. Ltd, Karnataka) and freeze dried MLE was stored at −4 °C refrigerated condition for further biochemical analysis.

##### 2.2.1.4 Microwave-assisted extraction method

Microwave-assisted extraction involved placing moringa leaf powder samples in a 70 % v/v hydroethanolic solvent system at a ratio of 1:10 within high-pressure TFM (tetrafluoroethylene) microwave cells of the Milestone Ethos™ X. Extraction of biomolecules from moringa leaves were effectuated at 600 W magnetron power for 30 min, according to the optimized procedure stated by Ref. [10]. After the extraction cycle, the leaf-solvent mixtures were cooled to room temperature and marc was subjected to repeated extraction for 3 times. Acquired extracts were separated, pooled and concentrated using a rotary evaporator (RV 3 Pro V, IKA India, Pvt. Ltd, Karnataka). Concentrated moringa leaf extracts were freeze-dried using Innova INOFD 12S, Innova Bio-Meditech, China and stored at refrigerated condition at −4 °C for ensuing analysis.

### 2.3 Characterization of extracted moringa leaf extract

#### 2.3.1 Phytochemical screening

The moringa leaf extract underwent a confirmatory phytochemical analysis to qualitatively identify alkaloids, flavonoids, steroids, reducing sugars, tannins, and saponins, following the procedures detailed by Ref. [11].

#### 2.3.2 Extraction yield

The extraction yield from the moringa leaves was determined by calculating the mass of lyophilized material obtained after complete solvent removal, relative to the mass of dried leaves used in the extraction process with the specified solvent system. The extraction yield for the moringa leaves was reckoned by the following formula(1):

(1) $$\text{Yield}\;\left(\%\right)=\frac{\text{Weight}\;\text{of}\;\text{the}\;\text{freeze}\;\text{dried}\;\text{extract}\;\text{acquired}\;\left(g\right)}{\text{Weight}\;\text{of}\;\text{the}\;\text{moringa}\;\text{leaf}\;\text{sample}\;\text{taken}\;\text{for}\;\text{extraction}\;\left(g\right)}\times 100$$

#### 2.3.3 Hygroscopicity

Hygroscopicity is the phenomenon of absorbing water molecules by the lyophilized moringa leaf extracts and also this ensures the stability of the biomolecules after extraction. Determination of hygroscopicity was followed by the protocol stated by Ref. [12]. In summary, lyophilized moringa extracts were placed on a pre-weighed Petri plate (W_1_) and positioned within a desiccator containing saturated sodium chloride solution on a ceramic stage at room temperature (25 ± 3 °C) (W_2_). Fourteen days later, the moringa samples were weighed (W_3_). Based on the following formula(2), the percentage of hygroscopicity was measured,

(2) $$\text{Hygroscopicity}\;\left(\%\right)=\frac{W_{3}‐W_{2}}{W_{2}‐\;W_{1}}\times 100$$

Where,

W_1_ = Mass of the empty Petri dish (g).

W_2_ = Mass of the Petri dish and MLE (Initial stage/Day 1) (g)

W_3_ = Mass of the Petri dish and MLE (After 14 days) (g)

#### 2.3.4 Total phenol content

The total phenol content of the lyophilized MLE was determined by the UV spectrophotometer method proposed by Ref. [13] with slight modifications. Lyophilized moringa leaf extracts were re-diluted in deionized water at a ratio of 1 mg/ml. To 100 μl of re-diluted moringa leaf extract, 400 μl of deionized water and 150 μl of FC reagents are added respectively. About 500 μl of 20 % sodium carbonate was added and kept under the dark for 1 h to develop a dark greenish-blue colour. 500 μl ethanol is used as blank. The colour change obtained in the mixture was measured at 650 nm using a Bio-Rad iMark™ Microplate Reader with 0.001 OD resolution controlled by Microplate Manager 6 software. The TPC in moringa extracts was estimated using the Gallic acid standard curve.

#### 2.3.5 Total flavonoid content

The total flavonoid content in the freeze-dried moringa leaf extract was determined using the aluminium chloride method following the suggested protocol of [13]. In a nutshell, the lyophilized leaf samples were re-diluted using deionized water similar to the TPC procedure. To the MLE sample solution, 10 % of 100 μl aluminium chloride solution and 1M sodium acetate is added and vortexed. After 45 min of incubation at room temperature, the development of yellow colour in the samples was measured using a Bio-Rad iMark™ Microplate Reader at 415 nm, with ethanol serving as the blank. The TFC in moringa extracts was estimated using the quercetin acid standard curve.

#### 2.3.6 Water solubility index

The solubility index of the moringa extracts was determined by dissolving the extract in distilled water and the solution was kept overnight in an oven at 80 °C poured on a pre-weighed aluminium pan [14]. The water solubility index was estimated using formula(3):

(3) $$\text{Water}\;\text{Solubility}\;\text{Index}\;\left(\%\right)=\frac{\text{Weight}\;\text{of}\;\text{dried}\;\text{moringa}\;\text{extract}\;solution (g)}{\text{Initial}\;\text{weight}\;\text{moringa}\;\text{extract}\;taken (g)}\times 100$$

#### 2.3.7 Protein content

The protein content in the lyophilized MLE was assessed using the modified biuret method for food samples as proposed by Nielsen in 2010. In outline, the freeze-dried moringa leaf extract of 10 mg was homogenized using 10 ml of phosphate buffer and centrifugated for 10 min at 10000 rpm. About 100 μl of supernatant solution and 400 μl biuret reagent are pipetted out in a 96-well microplate. The mixture was kept in a dark condition for 30 min and colour change was determined using iMark™ absorbance microplate reader (Bio-Rad laboratories) in three replications at OD 540 nm. The phosphate buffer without the addition of samples served as the blank, and the protein content (mg/ml) in the samples was determined using a standard graph of Bovine Serum Albumin (BSA).

#### 2.3.8 Ascorbic acid content

The ascorbic acid content in the moringa leaf extract was determined by the Oxalic Acid method [15]. Ascorbic acid content was quantified by titrating against the 2,6-Dichlorophenol Indophenol dye until the pink colour was obtained as an endpoint. The dye consumed during titration is directly pro rata to the amount of ascorbic acid present in the sample moringa extract solution. The ascorbic acid content is determined using formula (4)and values are expressed in mg/100g.

(4) $$\text{Ascorbic}\;\text{Acid}\;\left(mg/100g\right)=\frac{0.5\;\text{mg}}{V_{1}}\times \frac{V_{2}}{5\text{ml}}\times \frac{100\;\text{ml}}{W}\times 100$$

Where,

V_1_: Volume of dye consumed by the standard (L-Ascorbic Acid)

V_2_: Volume of the dye consumed by the moringa extract solution.

W: Weight of freeze-dried moringa leaf extract taken.

#### 2.3.9 Antioxidant activity (DPPH method)

The antioxidant activity of dried moringa leaf extract was determined by the DPPH method followed by Ref. [13] with minor modifications. In short, lyophilized moringa leaf extracts were diluted in 50 % methanol and mixed with 200 μl of 2,2′-Diphenyl-1-Picryl-Hydrazyl (DPPH) solution for every 600 μl of sample moringa extract solution. The mixture stored under dark conditions for 30 min is checked for colour change and its absorbance was determined at 517 nm using UV Spectrophotometer (iMark™ absorbance microplate reader, Mfg by Bio-Rad). Free Radical Scavenging activity was computed by following formula (5)and represented in percentage (%)

(5) $$\text{Radical}\;\text{Scavenging}\;\text{Activity}\;\left(\%\right)=\frac{A_{\text{Control}\;}‐\;A_{\text{Sample}}}{A_{\text{Control}}}\times 100$$

Where,

A_Control_: Optical Density at 517 nm (DPPH & Methanol)

A_Sample_: Optical Density at 517 nm (Lyophilized Moringa leaf extract)

#### 2.3.10 Phytonutrient profiling of moringa leaf extract by ICP-OES

The phytonutrient and elemental profiling of the lyophilized moringa leaf extract were identified using Inductively Coupled Plasma-Optical Emission Spectrometry (ICP-OES) (Prodigy High Dispersion ICP, Teledyne Leeman Labs, USA) calibrated with working standard solutions of 23 elements prepared by diluting in ultra-pure nitric acid as per the standardized procedure stated by Ref. [16]. In brief, 1 g of lyophilized moringa leaf extract was digested using 65 % nitric acid and hydrogen peroxide in 100 ml Pyrex® conical flask in a ratio of 4:1. The acid mixture is heated on a hot plate at 80 °C until a clear digested solution forms, then diluted with 100 ml of ultrapure deionized water. The experimental value obtained from the instrument was multiplied with a dilution factor (6)and expressed in mg/100g.

(6) $$\text{Dilution}\;\text{factor}=\frac{\text{Volume}\;\text{made}‐\text{up}\;\text{after}\;\text{digestion}\;\left(\text{ml}\right)}{\text{Weight}\;\text{of}\;\text{the}\;\text{sample}\;\text{taken}\;\left(g\right)}$$

#### 2.3.11 Surface morphology of moringa marc by Scanning Electron Microscope

The effect of intensified energy during the extraction procedure was investigated using the FEI Quanta™ 250 FEG model Scanning Electron Microscope equipped with EDAX detectors as per the protocol proposed by Ref. [17]. Briefly, 5 mg of lyophilized dried moringa marc was positioned on carbon stubs mounted on aluminium-made SEM stubs. The samples were coated with Au particles (gold) with 10 mm thickness using a mini plasma sputter coater and glow discharge system (Emitech Quorum SC7620, UK). High-resolution images were recorded at 1000× magnification and evaluated at 30 kv voltage under a high-vacuum environment throughout the electron column.

#### 2.3.12 HPTLC fingerprinting of quercetin in moringa leaf extracts

Initially, quercetin stock solutions were prepared by dissolving 10 mg of Sigma-Aldrich quercetin compound in 10 ml HPLC grade methanol and similarly, MO extracts were dissolved. Both solutions were purified using 0.45 μm bacteriological syringe filter. HPTLC fingerprinting procedure was followed as per the steps suggested by Ref. [18]. In short, the Mobile phase consisting of Toluene: Ethyl acetate: Chloroform: Formic Acid in the ratio 6:2:5:1.5 was prepared and sample solutions were loaded in silica gel TLC plates (60 F_254_ – Merck, Mumbai) using semi-automatic CAMAG© LINOMAT 5 applicator. The TLC plates with sample and standards were placed in CAMAG© twin trough containing the prepared mobile phase. Band development in TLC plates was identified by CAMAG© smartALERT and bands were studied using CAMAG© UV cabinet 4 at UV 254 nm. Quantification of quercetin in moringa extract was done using CAMAG© TLC Scanner 4 using VisionCAT software by generating densitometric graphical data. Photo-documentation of the TLC plates under a UV chamber was achieved by a Canon IXUS 285HS 20.2 MP digital camera.

### 2.4 Statistical analysis

The mean values of the respective parameters for freeze-dried moringa leaf extracts were analyzed using IBM SPSS Statistics (version 25.0). Results were presented as mean ± standard deviation for each extraction method. Experiments were conducted in triplicate using one-way ANOVA. The critical difference (CD) was calculated at a significance level of 5 % [19]. Graphs depicting the data were created using Origin Pro (Version 9.9).

## 3 Result and discussion

### 3.1 Proximate phytochemical assessment

The proximate assessment of the presence of phytochemicals such as alkaloid, flavonoid, saponin, sterols, tannins, and reducing sugars in the dried moringa leaf extract was detected for different extraction methods using 70 % v/v hydroethanolic solvent. The confirmatory presence of these secondary metabolites was tabulated in Suppl. Table 1.

Initial qualitative screening is valuable for identifying bioactive compounds, potentially contributing to drug development and manufacturing [20]. Phyto-alkaloid group act as a repellent to many insects along with wide microbiocidal attributes. Several alkaloids were also reported in the HIV & AIDS-related ailments [[21], [22], [23]]. Phyto-flavonoids were found to be mighty antioxidant activities in scavenging free radicals resulting in anticancer hallmarks [24]. Tannins from plant sources were reported to possess antiviral, antiparasitic, and antidiabetic activities and also help to maintain homeostasis of kidney functioning [25]. Saponins were a major ingredient as an adjuvant in the production of vaccines and were also reported to have properties such as anti-inflammatory, antiprotozoal, drug delivery adjuvant and anti-cholesterol [26].

All of these metabolites were found to be present in the extracts obtained from both conventional and non-conventional methods might be attributable to better solubility of these phytochemicals in the 70 % hydro-ethanolic solvent phase containing both polar and non-polar solvent combination as per the rule “*like dissolves like*” when compared to organic solvents alone [27,28]. In addition to this, the solubility of these metabolites might have increased due to a rise in temperature in all four extraction methods viz., Traditional/Maceration (E_1_), Soxhlet extraction (E_2_), Ultrasound Assisted Extraction method (E_3_), and Microwave Assisted Extraction method (E_4_). The findings were comparable to the results of [[29], [30], [31]] in moringa leaves respectively.

### 3.2 Extraction yield

The extraction yield of the MLE as a result of the extraction method showed the quantity of yield obtained during the procedure. Among the different extraction methods, extraction yield from ultrasound-assisted extraction (E_3_), showed a maximum recovery of 21.81 ± 0.32 % followed by microwave-assisted extraction (E_4_) (17.61 ± 0.45 %). The lowest recovery of moringa leaf extract was recorded in Soxhlet extraction (E_2_) (6.24 ± 0.08 %). The higher extraction yield recovery in the non-conventional extraction methods such as E_3_ & E_4_ was influenced by both extraction methods as well as the solvent phase system (Table 1). Maximum recovery in UAE could be attributed to the effects of ultrasound waves produced from the probe of the instrument acting directly on the surface of the dried moringa leaf powder under the hydroethanolic solvent phase which is potentially involved in the disruption of cell wall structure. Similarly, the microwaves and the thermal energy generated by the magnetron disrupted the cellular integrity of moringa leaves immersed in the solvent during microwave-assisted extraction, contrasting with outcomes from traditional extraction methods. The research findings were in parallel to the results of [[32], [33], [34]] from moringa leaves with extraction yield of 19.92 %, 32.77 % and 34.94 %, respectively.

**Table 1:** Comparative analysis of phyto-molecule levels in moringa leaf extracts from various extraction methods.

Phyto-Molecules	Extraction Methods				SE(d)	Cd (5 %)
	E_1_	E_2_	E_3_	E_4_		
Extraction Yield (%)	15.61 ± 0.2^c^	6.24 ± 0.80^d^	21.81 ± 0.32^a^	17.61 ± 0.45^b^	0.21	0.47
Total Phenol content (mg GAE/g)	131.33 ± 0.8^c^	126.74 ± 1.72^d^	148.86 ± 1.92^a^	137.65 ± 4.49^b^	1.85	4.04
Total Flavonoid content (mg QE/g)	18.69 ± 0.49^c^	17.04 ± 0.38^d^	23.18 ± 0.44^a^	22.12 ± 0.61^b^	0.34	0.75
Protein content (mg/ml)	131.11 ± 2.40^c^	121.39 ± 0.66^d^	148.66 ± 3.74^a^	139.64 ± 1.71^b^	1.70	3.70
Ascorbic acid content (mg/100g)	466.32 ± 0.60^c^	358.41 ± 1.94^d^	620.25 ± 1.4^a^	535.33 ± 3.5^b^	7.59	16.55
Antioxidant activity (%)	78.47 ± 2.08^a^	73.39 ± 2.04^d^	86.25 ± 1.58^a^	81.98 ± 1.72^b^	1.32	2.88
IC_50_ (μg/ml)	24.59 ± 2.08^c^	33.31 ± 2.04^d^	11.62 ± 1.58^a^	14.55 ± 1.72^b^	0.28	0.61
Solubility (%)	96.74 ± 0.13	95.92 ± 0.71	96.24 ± 0.47	95.90 ± 0.13	0.69	NS
Hygroscopicity (%)	43.55 ± 0.67	43.68 ± 0.16	43.42 ± 0.31	43.26 ± 0.23	7.17	NS

∗NS (Non-significant).

∗∗(E_1_ – Maceration Extraction; E_2_ – Soxhlet Extraction; E_3_ – Ultrasound Assisted Extraction; E_4_ - Microwave Assisted Extraction).

### 3.3 Hygroscopicity

Hygroscopicity refers to the capacity of a substance to adsorb moisture from its surroundings and also decides the stability, flowability and surface properties. Hygroscopicity of the dried moringa extract from both conventional and non-conventional extraction methods was not significant (Table 1), almost all the extracts showed high hygroscopicity ranging from 43.26 ± 0.23 % to 43.68 ± 0.16 %. The maximum hygroscopic nature of the freeze-dried moringa leaf extracts could be due to the leaching of various phytochemicals such as enzymes, cellulose, hemicellulose, carbohydrates, fatty acids, pectin, organic acids, proteins, glycosides, lignin, etc. [35]. The dried moringa leaf extract complex might be consisting of carbohydrates which could make extract matrix to get high affinity towards the surrounding water molecules. Usually, dried plant extracts are amorphous in nature and crystallization is hindered kinetically due to the molecular motion [36,37].Similarly, high hygroscopicity of plant extract after the extraction procedure was reported by Ref. [38] in moringa [39],in *Aloe vera* [40], in *Centella* sp. and [41] in garden heliotrope.

### 3.4 Total phenol and flavonoid content

The extraction methods exhibited notable variations in the phenol and flavonoid content of the moringa leaf extract (Table 1). Maximum phenol and flavonoid content were obtained in leaf extracts from UAE (148.86 ± 1.92 mg GAE/g and 23.18 ± 0.44 mg QE/g) followed by MAE (137.65 ± 4.49 mg GAE/g and 22.12 ± 0.61 mg QE/g). The lowest values of total phenol and flavonoid content were recorded in the conventional method of extraction – Soxhlet (E_2_) (126.74 ± 1.92 mg GAE/g and 17.04 ± 0.38 mg QE/g). High recovery of phenol and flavonoid contents from moringa leaves was mainly due to the increased magnitude of ultrasound waves during the instrumental operation resulting in sonolysis of cell wall by cavitation phenomena leading to leaching of phytochemicals into the solvent system [9]. Correspondingly, rehydrated moringa leaf cells under the hydroethanolic phase get expanded and Cell disruption transpired during microwave-assisted extraction through the interaction of microwaves with water molecules encompassing the leaf fragments [42]. The results of increased recovery of TPC and TFC were analogous to the recorded findings of [7,34,[43], [44], [45]] and [46] in *Moringa* sp. and rugosa rose respectively.

### 3.5 Protein content

Different extraction methods showed significant variations in the protein content present in the lyophilized moringa leaf extract. When juxtaposed with conventional extraction methods, Ultrasound-assisted extraction methods showed maximum recovery of protein 148.66 ± 3.74 mg/ml followed by microwave-assisted extraction method (139.64 ± 1.71 mg/ml) (Table 1). Maximum protein recovery in UAE and MAE might be attributed to continuous reverberations of ultrasound waves and emanation of microwaves on the moringa leaf samples under 70 % v/v hydro-alcoholic phase [47,48] respectively, this could have damaged the moringa leaf cellular matrix and enhanced the solubility of protein compounds. The findings were endorsed by the results of [49]in moringa leaves [50],in potatoes using ultrasonication [51], in milk-cap mushrooms using MAE and [52] in peas using UAE.

### 3.6 Ascorbic acid content

The freeze-dried moringa leaf extract exhibited significant changes in the ascorbic acid content among different extraction methods (Table 1). Ascorbic acid content was found to be ranging from 358.41 ± 1.94 mg/100g to 620.25 ± 1.42 mg/100g. Maximum ascorbic acid was recovered in the extracts obtained from UAE (E_3_) followed by MAE (E_4_). The lowest recovery of the ascorbic acid from the Soxhlet method (E_2_) was mainly due to exposure to high temperature for a prolonged period during the siphoning cycles. In contrast, UAE and MAE recorded increased ascorbic acid recovery due to ultrasonication and microwave radiation for a lesser duration not exceeding 30 min of extraction time. The impact of ultrasound and microwave energy through these non-conventional extraction methods ruptured moringa leaf cells greatly than conventional methods. Similar results were reported by various researchers and their findings are in corroboration with [9,53,54]in moringa respectively.

### 3.7 Antioxidant activity (DPPH method) and half-maximal inhibitory concentration (IC_50_)

The radical scavenging activity of the moringa leaf extract was determined by DPPH method for all extraction methods. The antioxidant group of compounds recovered from the moringa leaves showed better significant scavenging properties (Table 1). DPPH assay revealed that maximum antioxidant activity of 86.25 ± 1.58 % was observed in MO extracts from UAE (E_3_) subsequent to 81.98 ± 1.72 % in MAE (E_4_). The highest RSA of the MLE obtained through UAE and MAE modus operandi might be attributed to the drying technique of the moringa leaf sample using solar cabinet dryer at 40 ± 2 °C [55]. Additionally, combined effects of ultrasonication and microwave radiation on MO leaves under 70 % v/v hydroethanolic solvent system promoted shearing of moringa plant cells within very short period of time at lower extraction time compared to maceration method (E_1_) (78.47 ± 2.08 %) and Soxhlet (E_2_) (73.39 ± 2.04 %).

The antioxidant efficacy of the MLE derived from different extraction methods was assessed via the DPPH assay to ascertain the half-maximal inhibitory concentration (IC50).The half-maximal inhibitory concentration values exhibited significant variations among the moringa leaf extracts obtained through different extraction methods. The minimal concentration required to scavenge 50 % of the free radicals was observed with UAE. with an IC50 of 11.62 ± 1.58 μg/ml followed by 14.55 ± 1.72 μg/ml in MAE (E_4_), whereas the highest IC50 value was recorded in the Soxhlet extraction method (E_2_) 33.31 ± 2.04 μg/ml. The superior half-maximal inhibitory activity of MO leaf extract might be due to the maximum leaching of phyto-molecules into the solvent system as a result of the sonolysis of moringa cellular matrix. The recorded data of higher antioxidant activity and half-maximal inhibitory activity was substantiated with the results of [9,29,53,[56], [57], [58]]within moringa, respectively.

### 3.8 Phytonutrient profiling of moringa leaf extract by ICP-OES

About 23 phytonutrients were screened in lyophilized MO leaf extracts obtained using conventional and non-conventional methods. The screened nutrients were quantified through Inductively Coupled Plasma Optical Emission Spectroscopy (ICP-OES) by digesting in acid (Table 2 and Suppl. Fig. 1). Among the various extraction methods, the nutrient content of the leaf extract demonstrated significant differences in the UAE (E_3_) and MAE (E_4_) when compared to conventional extraction methods (E_2_ and E_1_). Observed 23 phytonutrients *viz*. aluminium, cadmium, calcium, chromium, copper, ferrous, lead, magnesium, manganese, molybdenum, phosphorus, potassium, zinc, nickel, boron, sodium, silver, barium, bismuth, cobalt, gallium, indium and strontium were reported from instrumental results. Among 23 elements some important elements like Fe, Zn, Ca, Mg, Mn, K, P and Na were obligatory for the development and growth of both plants and human beings.

**Table 2:** ICP-OES quantitative evaluation of 23 phyto-minerals in moringa leaf extract.

Elements (mg/100g)	Extraction Methods				SE(d)	Cd (5 %)
	E1	E2	E3	E4		
Al	59.26 ± 0.83	51.83 ± 0.38	64.70 ± 1.49	61.05 ± 0.91	7.783	NS
Cd	4.55 ± 0.064^b^	4.35 ± 0.041^c^	5.15 ± 0.157^a^	4.64 ± 0.034^b^	0.076	0.165
Ca	1810.07 ± 41.59^ab^	1729.99 ± 38.84^c^	1843.96 ± 20.07^a^	1787.92 ± 30.41^b^	24.06	52.43
Cr	44.46 ± 0.99^a^	41.64 ± 0.14^b^	45.41 ± 0.77^a^	45.16 ± 0.21^a^	0.850	1.853
Cu	28.79 ± 0.49^bc^	27.58 ± 0.05^c^	30.26 ± 0.98^a^	29.41 ± 0.80^ab^	0.60	1.32
Fe	290.32 ± 6.32^d^	329.69 ± 10.54^c^	504.72 ± 5.83^a^	419.98 ± 7.71^b^	5.02	10.94
Pb	8.29 ± 0.13^b^	6.78 ± 0.12^c^	8.79 ± 0.14^a^	8.65 ± 0.21^a^	0.12	0.26
Mg	2552.00 ± 51.12^a^	2351.18 ± 44.79^b^	2571.55 ± 15.74^a^	2545.15 ± 31.17^a^	26.07	56.80
Mn	54.13 ± 0.19^b^	50.80 ± 1.48^c^	56.83 ± 0.38^a^	55.22 ± 0.03^b^	0.57	1.24
Mo	11472.76 ± 263.65^a^	9419.34 ± 288.40^b^	11480.39 ± 117.17^a^	11457.41 ± 241.66^a^	131.49	286.51
P	84.48 ± 2.24^b^	79.44 ± 0.86^c^	89.17 ± 2.00^a^	88.57 ± 0.36^a^	0.94	2.06
K	6639.98 ± 164.33^b^	5956.14 ± 129.68^c^	6866.46 ± 154.17^a^	6774.92 ± 23.04^a^	53.21	115.95
Zn	49.61 ± 1.14^b^	37.88 ± 0.30^c^	52.34 ± 1.10^a^	51.66 ± 0.42^a^	0.68	1.49
Ni	22.59 ± 0.49^a^	19.55 ± 0.46^b^	22.73 ± 0.69^a^	22.64 ± 0.46^a^	0.26	0.57
B	143.88 ± 4.15^a^	137.19 ± 2.05^b^	147.05 ± 2.50_a_	145.26 ± 4.44^a^	1.96	4.29
Na	225.55 ± 4.12	224.48 ± 5.34	226.66 ± 5.55	226.51 ± 2.62	3.01	NS
Ag	275.08 ± 1.94	284.09 ± 5.79	286.72 ± 0.39	285.21 ± 0.38	4.72	NS
Ba	8.33 ± 0.10	8.38 ± 0.14	8.51 ± 0.17	8.41 ± 0.18	0.09	NS
Bi	8.15 ± 0.06^b^	7.23 ± 0.10^c^	9.28 ± 0.01^a^	8.39 ± 0.07^b^	0.14	0.30
Co	1.50 ± 0.036^b^	1.53 ± 0.024^b^	1.67 ± 0.03^a^	1.55 ± 0.01^b^	0.02	0.05
Ga	116.01 ± 1.57^ab^	109.12 ± 0.29^c^	113.07 ± 2.76^b^	119.16 ± 1.21^a^	165.64	360.90
In	16.56 ± 0.15^c^	16.32 ± 0.04^c^	18.95 ± 0.14^b^	22.84 ± 0.60^a^	0.33	0.73
Sr	287.34 ± 1.86	278.95 ± 6.64	291.42 ± 0.99	293.21 ± 5.78	5.18	NS

∗ NS: Non-significant.

∗∗(E_1_ – Maceration Extraction; E_2_ – Soxhlet Extraction; E_3_ – Ultrasound Assisted Extraction; E_4_ - Microwave Assisted Extraction).

These mineral elements play essential roles in cellular function, serving as structural components of tissues, constituents of bodily fluids, and integral components of enzymes crucial to major metabolic pathways. In addition, moringa leaves consist of macronutrients required for survival. These nutrients play a critical role in the body, functioning as cofactors or carriers and participating in the metabolic processes involved in the breakdown and utilization of macronutrients.Various parts of the Moringa plant are recognized as excellent sources of essential minerals, such as calcium (Ca), phosphorus (P), manganese (Mn), zinc (Zn), and chromium (Cr). Moreover, the leaves and flowers present a promising potential as natural sources of iron (Fe) supplements for human consumption [[59], [60], [61]]. Also, these phyto-minerals in moringa leaf extract, when consumed by human beings as a food supplement or in other ways may help to alleviate malnutrition efficaciously.

Almost many phyto-minerals were found to be quantified at higher concentrations in the extracts from UAE (E_3_) followed by MAE (E_4_). This is mainly due to the cavitation principle that occurred during the sonication process, where the explosion of water bubbles produced in hydroethanolic media (70 % v/v) by the probe led to the rupturing of moringa leaf cells and leaching of the phyto-components along with nutrients would have taken place [62,63]. Similarly, the microwave radiation interacted with the rehydrated leaves resulting in the expansion and bursting of plant cells resulting in exuding phyto-constituents into the solvent by vibrating the water molecules (*i.e*., dipolar rotation) at a faster rate creating continuous collision with surrounded molecules converting into thermal energy rapidly near the powdered leaf samples [42,64]. Similarly, nutrient screening and phyto-nutrient analysis results correlated with the findings of [[65], [66], [67], [68], [69], [70], [71], [72]] in moringa leaves, respectively. The presence of these elements in the plants and the benefits of consuming these elements by humans through a plant-based diet were reported in Suppl. Table 2 [[73], [74], [75], [76], [77], [78], [79], [80], [81], [82], [83], [84], [85], [86], [87], [88], [89], [90]].

### 3.9 Quercetin content

**Fig. 1:**
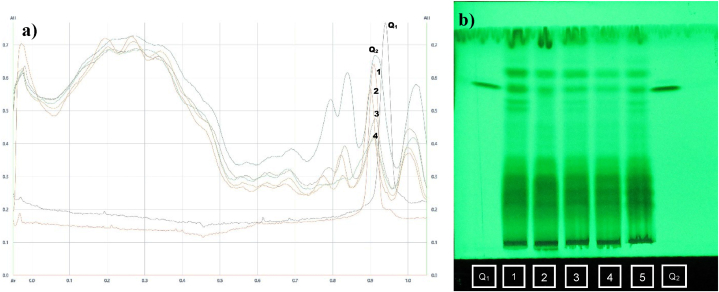
a) HPTLC densitogram of Quercetin in Moringa extract (Red colour) and b) HPTLC Chromatogram of *Moringa oleifera* extract from different extraction methods. ∗Lane Q_1_ and Q_2_: Bands formed byQuercetin standard (10 μl and 20 μl);**Lane1:** Moringa extract of UAE; **Lane2:** Moringa extract of MAE; **Lane3:** Moringa extract of Soxhlet extraction and **Lane5:** Moringa extract of Maceration extraction (Non-adjusted raw images were submitted in the supporting supplementary material as Suppl. Figs. 2 and 3).

Moringa leaf extracts obtained through various extraction methodologies underwent GC-MS profiling, and identified compounds were documented in previously published research paper of [91]. Among many compounds, quercetin, kaempferol and myricetin were found to be unique and higher quantity in moringa leaves [92]. The presence of quercetin alone was identified and quantified using the HPTLC fingerprinting method(Fig. 1b). Levels of quercetin were quantified (Fig. 1a)to be high in UAE (674.70 μg/ml) followed by MAE (480.80 μg/ml). Maximized quercetin levels were due to the sonolysis of moringa leaf cells during the extraction procedure [93]. The lowest quercetin levels were recorded from Soxhlet (273.60 μg/ml) might be due to its temperature sensitivity exposed to prolonged extraction time.

### 3.10 Effect of extraction methods on moringa leaf residue by Scanning Electron Microscopy method

**Fig. 2:**
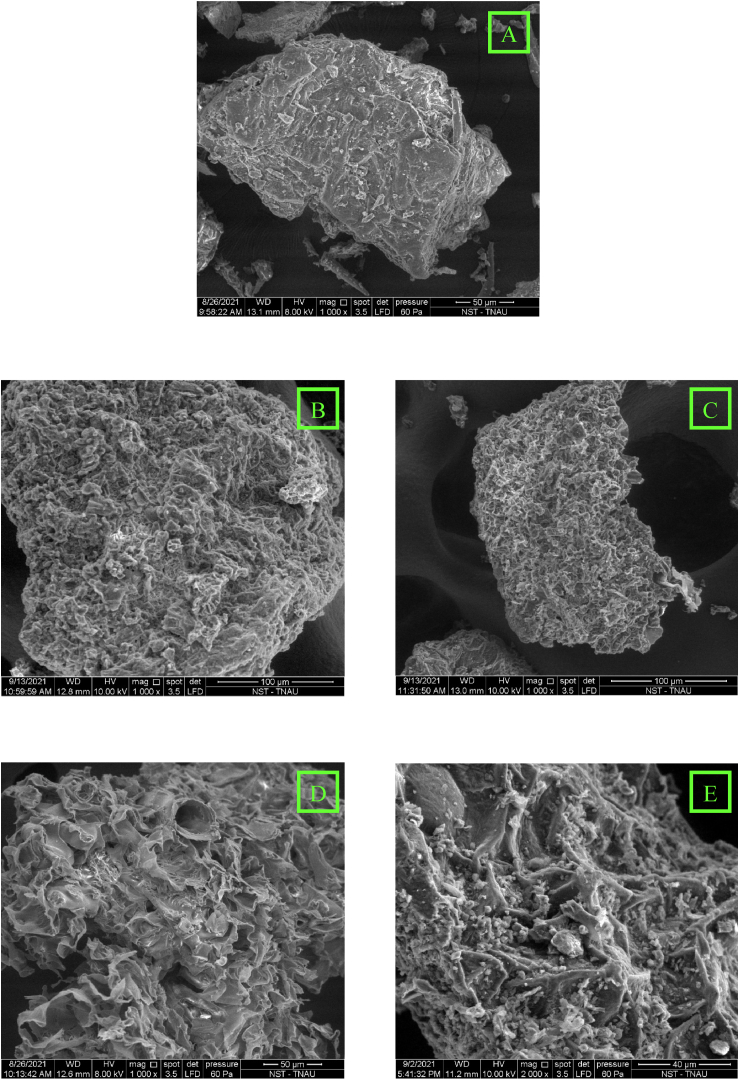
Scanning Electron Microscopy (SEM) images showing: [A] Moringa leaf sample (control), [B] Moringa marc after maceration, [C] Moringa marc after Soxhlet extraction, [D] Moringa marc after ultrasound-assisted extraction and [E] Moringa marc after microwave-assisted extraction.

The recovery of phytochemicals and other biochemical compounds from drumstick leaves was enhanced through the continuous application of energy sources, including ultrasonication, microwave radiation, and thermal energy, directed onto the sample surface. In order to study the morphological transformations that occurred during the extraction procedure, the moringa leaf sample residues were collected and studied through a Scanning Electron Microscope (SEM). Significant visual differences in the intensity of damages on the moringa leaf sample surfaces were recorded using SEM micrographs (Fig. 2). The SEM images of moringa marc obtained from four different extraction methods were compared with the grounded moringa leaf samples (control) (Fig. 2a). Characteristic depth of penetration by ultrasonicated waves and microwave radiation along with increased temperature in E_3_ and E_4_ extraction methods were studied respectively. Each energy source affects the efficiency of compound extraction, with ultrasonication providing mechanical disruption of plant cells and microwaves generating localized heating.Both methods are designed to enhance solvent penetration and facilitate the release of bioactive compounds. These extraction methods, particularly the influence of temperature, plays a crucial role in optimizing extraction yields and improving the recovery of phytochemicals.SEM micrographs of UAE (Fig. 2d) and MAE (Fig. 2e) revealed crucial disruptions and multitudinous hollow orifices affected by cavitation phenomena in UAE and by rapid dipolar oscillation-collision of water molecules around the plant samples in MAE, respectively.

Similar disruptions in the moringa leaf samples were observed in both the maceration (Fig. 2b) and Soxhlet extraction (Fig. 2c) methods. These disruptions are likely attributed to cellular imbibition and swelling, which occurred due to the prolonged soaking of the plant material, combined with the application of thermal energy. In maceration, extended contact between the solvent and plant tissue facilitates the absorption of the solvent by the cells, causing them to swell and rupture, allowing the release of phytochemicals. Similarly, in Soxhlet extraction, the repeated heating and cooling cycles, along with sustained solvent exposure, lead to a comparable effect, enhancing cell wall permeability and promoting the extraction of bioactive compounds. These phenomena highlight the importance of both solvent interaction time and temperature in maximizing extraction efficiency through mechanical and thermal disruption of plant cell structures. As a result of the intensified impact of energy on the imbibed leaf surface, the phytomolecules in the cells were leached out resulting in maximum recovery of biomolecules and phytonutrients [94,95]. These observations align with the findings of Ultrasound-Assisted Extraction in *Moringa* sp. reported by Refs. [34,45].

## 4 Conclusion

Recently, non-conventional extraction methods have garnered increasing attention due to their eco-friendliness, rapid processing capabilities, and improved qualitative yields. In contrast, conventional methods tend to be simpler and more time-consuming, often resulting in lower qualitative outcomes. From the proximate phytochemical analysis and phytonutrient screening, it was found that moringa leaf extracts are as promising as natural moringa leaves in a concentrated form. The findings from this research indicate that employing a 70 % v/v hydroethanolic solvent phase with ultrasound-assisted extraction (UAE) and microwave-assisted extraction (MAE) significantly enhanced the recovery of phytocompounds from Moringa leaf samples. The specific recovery amounts were as follows: protein (148.66 mg/100g and 139.64 mg/100g), Total Phenolic Content (148.68 mg GAE/g and 137.65 mg GAE/g), Total Flavonoid Content (23.18 mg QE/g and 22.12 mg QE/g), ascorbic acid (620.25 mg/100g and 535.33 mg/100g), Reactive Scavenging Activity (86.25 % and 81.98 %), and phyto-minerals (including Fe, Zn, Ca, Mg, K, P, Cu, Mn, Na). These results demonstrate that the enhanced energy input from ultrasonication and microwave radiation significantly facilitates the extraction process compared to conventional methods, allowing for substantial recovery in a considerably shorter timeframe. These innovative extraction techniques represent a major advancement for extraction-based industries, enabling the efficient acquisition of large quantities of phytomolecules. Notably, UAE exhibited a more rapid extraction process than MAE, achieving a higher recovery rate of 21.81 % within only 10 min.

However, the obtained moringa leaf extract was highly hygroscopic, presenting challenges in handling and stability. Despite this, the rapidly recovered biomolecules and phytonutrients from these novel methodologies could be effectively utilized in the development of specialized nutraceuticals and biofortified food products. Future research should prioritize stabilizing the hygroscopic moringa leaf extracts through encapsulation using edible food-based polymers. Such innovative food products will play a crucial role in modern nutrition by promoting healthy eating and addressing malnutrition.

## CRediT authorship contribution statement

**Arumugam Thangaiah:** Writing – original draft, Validation. **Sandeep Gunalan:** Writing – review & editing, Formal analysis, Data curation. **Premalakshmi Velu:** Supervision, Resources, Funding acquisition. **Dheebisha Chandirasekaran:** Methodology. **Aruliah Rajasekar:** Validation, Investigation, Data curation. **Mohamad S. AlSalhi:** Supervision, Funding acquisition. **Sandhanasamy Devanesan:** Methodology, Conceptualization. **Tabarak Malik:** Supervision, Resources.

## Data availability statement

All data generated or analyzed during this study are included in this published article. No additional data are available beyond what has been presented in the manuscript and its supplementary materials.

## Declaration of generative AI and AI-assisted technologies in the writing process

No artificial intelligence (AI) or AI-assisted technologies were used in the development, analysis, or preparation of this manuscript.

## Declaration of competing interest

The authors declare that they have no known competing financial interestsor personal relationships that could have appeared to influence the work reported in this paper.
