# Supplementary material for: Augmenting phyto-chemical and phyto-mineral profiling of moringa leaf extract: A contrastive study of solid-liquid extraction methodologies
Source: Heliyon. 2024 Dec 4;10(24):e40909. doi: 10.1016/j.heliyon.2024.e40909 (PMC11700248; doi:10.1016/j.heliyon.2024.e40909)
Supplement: Multimedia component 1 [file mmc1.docx]

**Suppl. Table 1. Screening of phyto-compounds in moringa leaf extracts using various extraction methods**

| **Phyto-compounds** | **Test/experiment** | **Blank** | **Hydro-ethanolic extract** | | | |
| --- | --- | --- | --- | --- | --- | --- |
|  |  |  | **E_1_** | **E_2_** | **E_3_** | **E_4_** |
| **Alkaloids** | Wagner’s test | **-** | **+** | **+** | **++++** | **++++** |
| **Flavonoid** | Lead acetate | **-** | **++** | **++** | **+++** | **+++** |
| **Saponin** | Foam test | **-** | **+** | **+** | **++** | **++** |
| **Sterols** | Liebermann- Burchard’s test | **-** | **+** | **+** | **+** | **+** |
| **Tannins** | Ferric salt test | **-** | **+** | **+** | **+** | **+** |
| **Reducing sugars** | Fehlings test | **-** | **++** | **+** | **+++** | **+++** |
| **Oils and fats** | Copper sulphate | **-** | **+++** | **++** | **+++** | **+++** |
| **Protein** | Biuret test | **-** | **+++** | **++** | **++++** | **++++** |
| **Carbohydrate** | Molisch’s test | **-** | **+++** | **++** | **++++** | **++++** |
| **Amino Acid** | Millons’s test | **-** | **++** | **+** | **+++** | **+++** |
| **Triterpenoids** | Salkowski’s test | **-** | **+++** | **++** | **++++** | **++++** |

*** - (Nil) ; + (Low); ++ (Medium); +++ (High); ++++ (Maximum)**

**(E_1_ – Maceration Extraction; E_2_ – Soxhlet Extraction; E_3_ – Ultrasound Assisted Extraction; E_4_ - Microwave Assisted Extraction)

**Suppl. Table 2. Roles and Benefits of Phytonutrients in Plants and Human Health**

| **S. No** | **Element** | **Role of the elements in** | |  |
| --- | --- | --- | --- | --- |
|  |  | **Plants** | **Human beings** | **Reference** |
|  | Iron (Fe) | An integral component of chlorophyll pigment, Electron transport chain | Functioning of the central nervous system, Haemoglobin synthesis | [73]; [74]  (Karim et al., 2024) |
|  | Zinc (Zn) | ribosomal fractions, cytochrome synthesis, carbonic anhydrase, gene expression, tolerance to abiotic stress, protein synthesis | Gene transcription, Protein synthesis, DNA synthesis, mitosis | [75] ; [76] |
|  | Calcium (Ca) | Structural framework for cell wall, enzyme activation and cellular signalling | Abundant mineral in bones & teeth, vasodilation, intercellular signalling, hormonal secretion | [77]; [5] |
|  | Magnesium (Mg) | Chlorophyll synthesis, assimilation of photosynthates enzyme activation, phosphate metabolism and ATP synthesis | RNA, DNA, protein synthesis, regulate inter-cellular levels of calcium and potassium, nerve transmission, bone turnover, vasomotor tone and cardiac excitability | [78] ; [79] |
|  | Potassium (K) | Transportation of carbohydrates into cells assisting in ATP production, | regulates acid-base levels, triggers electrical impulses for both muscle and nerve cells and maintains blood pressure | [80] ; [81]; [82] ; [83] |
|  | Phosphorus (P) | Formation of DNA and RNA structure by phosphate bonds, | involves in complex physiological responses and acts as a signalling molecule |  |
|  | Copper (Cu) | Chlorophyll and seed production, cofactors for enzymatic activities, act as protein trafficking machine and iron mobilization | Bone health, immune function and cholesterol metabolism | [84] ; [85]; [86] |
|  | Manganese (Mn) | Cofactor for metalloenzyme production, enzyme-catalytic reactions | regulate glucose and lipid metabolism, scavenge free radicals in mitochondria, enzyme synthesis, improve the immune system | [87] ; [88] |
|  | Sodium (Na) | Maintain osmotic pressure, turgidity of cells, | Essential electrolyte, blood sodium level, activates renin-angiotensin–aldosterone system (RAAS). | [89]; [90] |

**
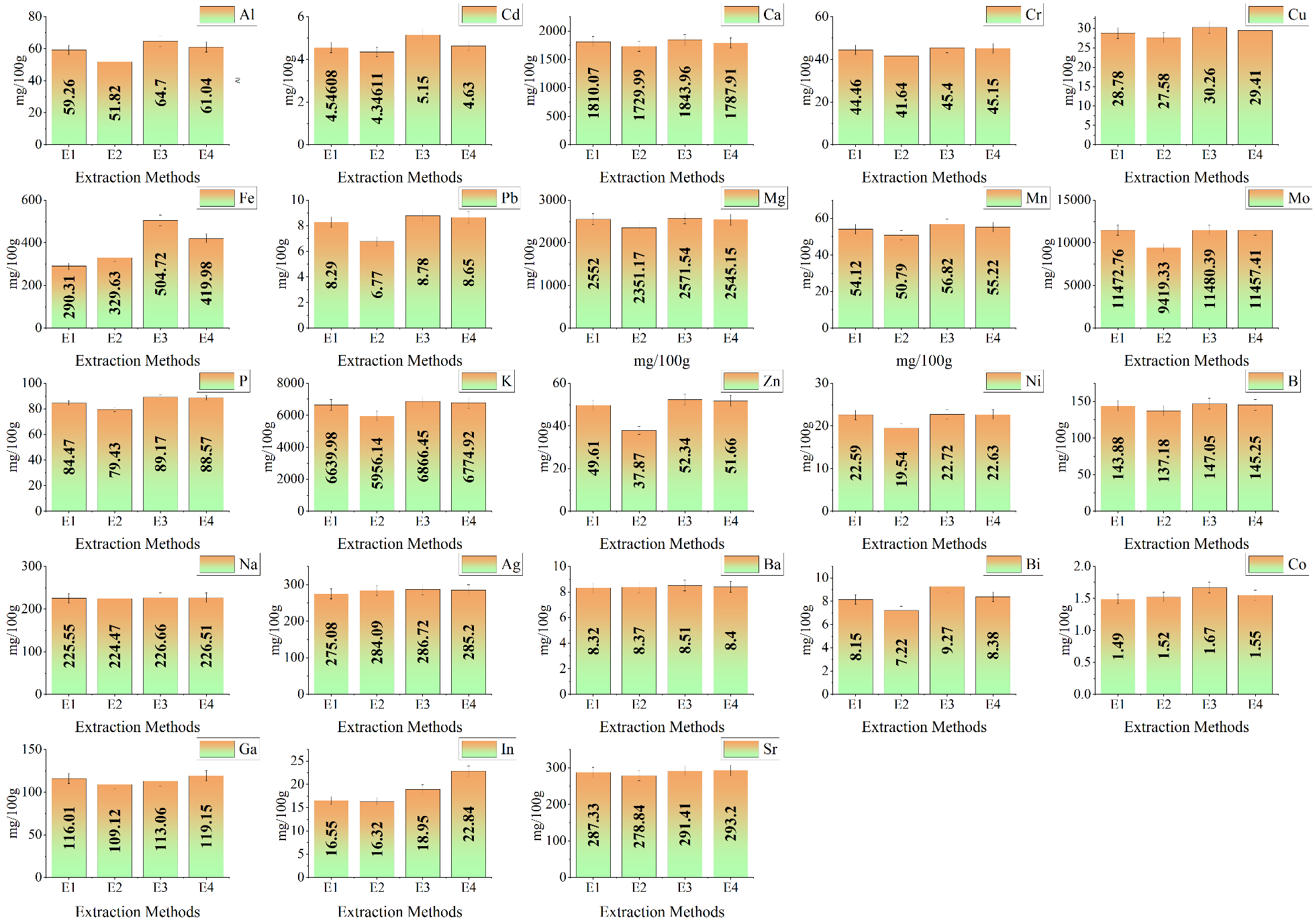
Suppl. Figure 1. Quantitative screening and analysis of 23 phyto-minerals present in moringa leaf extract by ICP-OES**

**(E_1_ – Maceration Extraction; E_2_ – Soxhlet Extraction; E_3_ – Ultrasound Assisted Extraction; E_4_ - Microwave Assisted Extraction


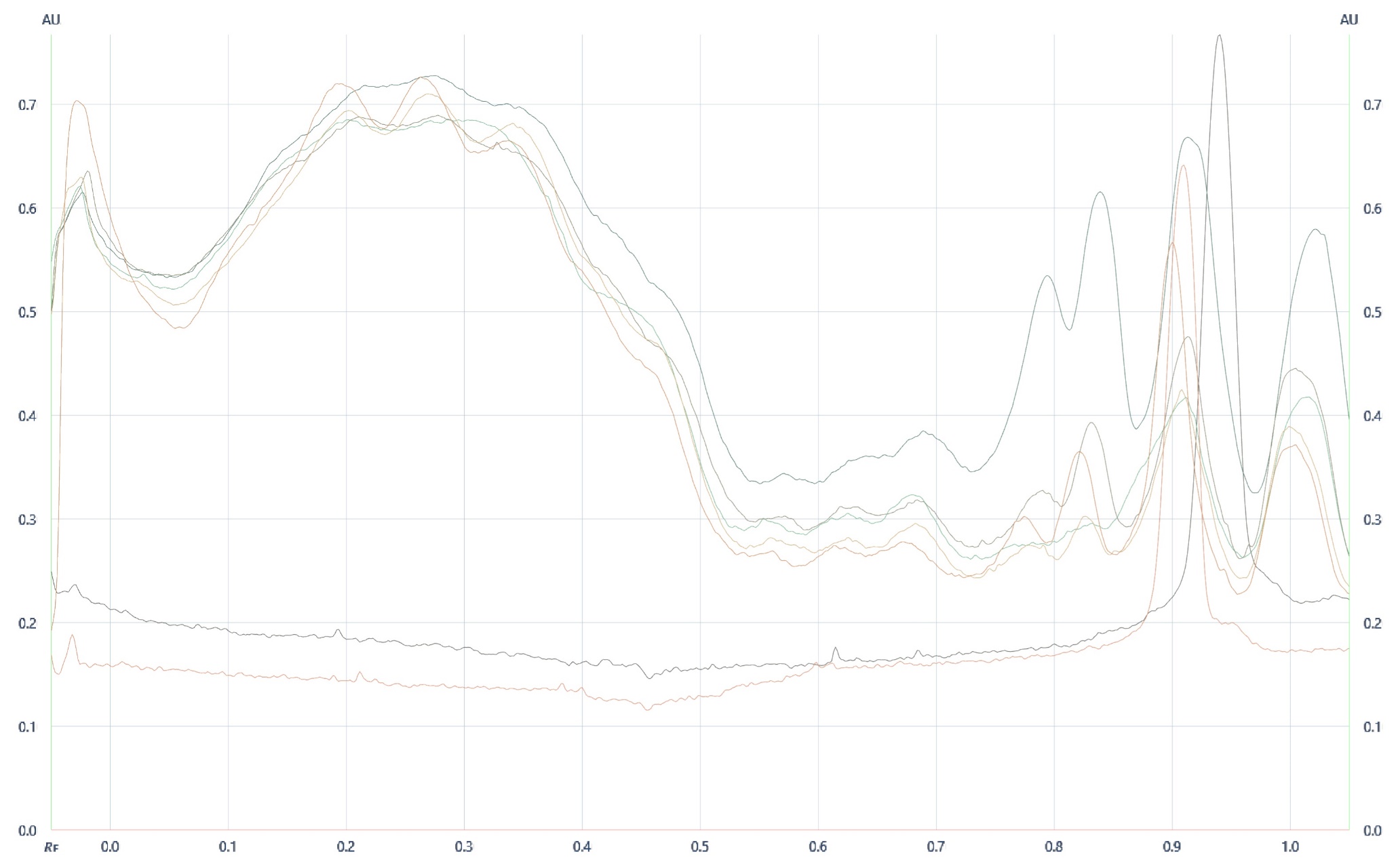


**Suppl. Figure 2. HPTLC densitogram of Quercetin in in Moringa leaf extract from different extraction methods (Red colour)**


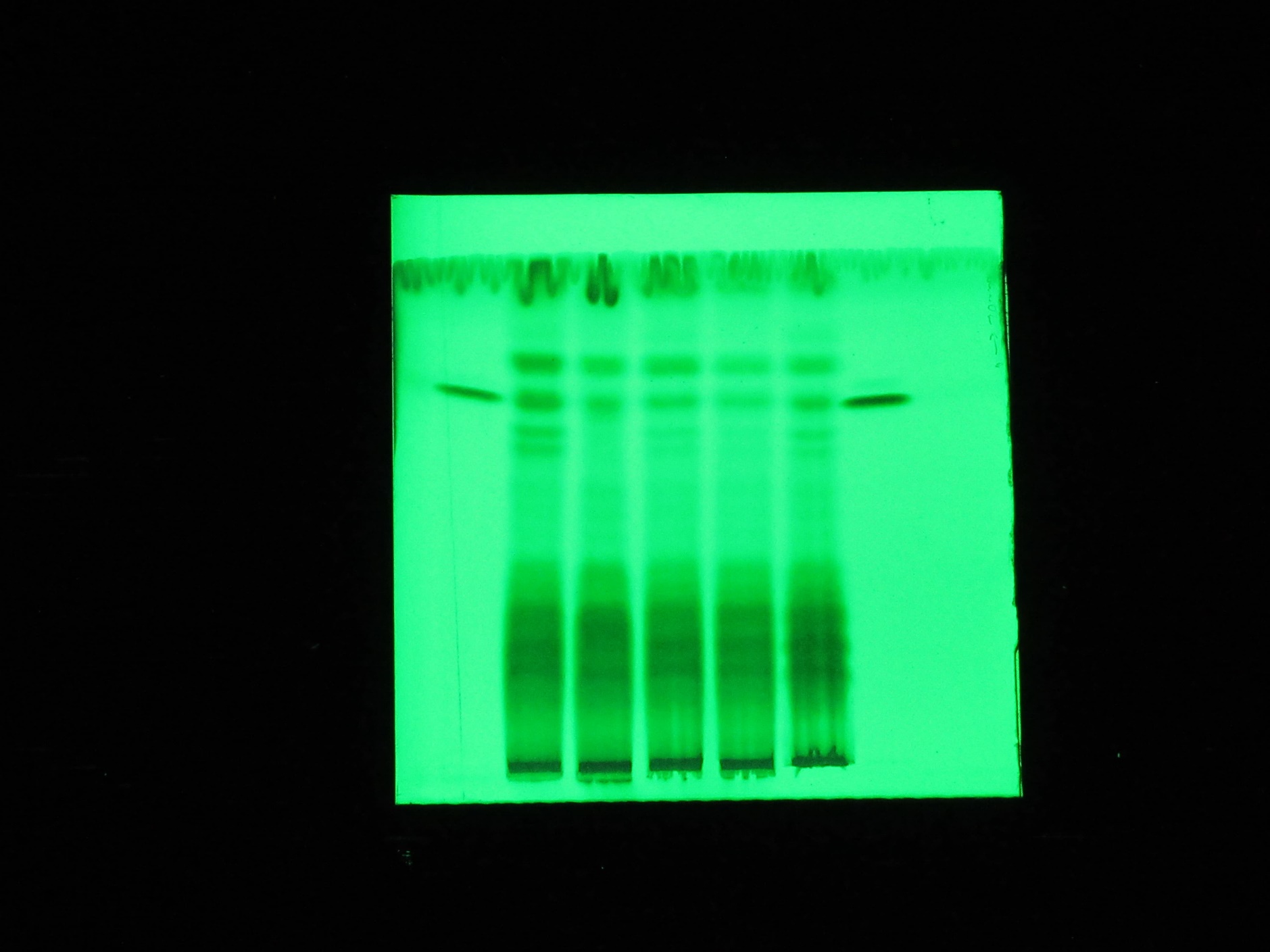


**Suppl. Figure 3. HPTLC Chromatogram of Quercetin in Moringa leaf extract from different extraction methods**
